# Mobile phone use and glioma risk: A systematic review and meta-analysis

**DOI:** 10.1371/journal.pone.0175136

**Published:** 2017-05-04

**Authors:** Ming Yang, WenWen Guo, ChunSheng Yang, JianQin Tang, Qian Huang, ShouXin Feng, AiJun Jiang, XiFeng Xu, Guan Jiang

**Affiliations:** 1 Department of Stereotactic Radiosurgery, Affiliated Hospital of Xuzhou Medical University, Xuzhou, China; 2 Department of Radiotherapy, Affiliated Hospital of Xuzhou Medical University, Xuzhou, China; 3 Department of Dermatology, the Affiliated Huai’an Hospital of Xuzhou Medical University, Huai’an, China; 4 Department of Dermatology, Affiliated Hospital of Xuzhou Medical University, Xuzhou, China; Ohio State University Wexner Medical Center, UNITED STATES

## Abstract

**Objective:**

Many studies have previously investigated the potential association between mobile phone use and the risk of glioma. However, results from these individual studies are inconclusive and controversial. The objective of our study was to investigate the potential association between mobile phone use and subsequent glioma risk using meta-analysis.

**Methods:**

We performed a systematic search of the Science Citation Index Embase and PubMed databases for studies reporting relevant data on mobile phone use and glioma in 1980–2016. The data were extracted and measured in terms of the odds ratio (OR) and 95% confidence interval (CI) using the random effects model. Subgroup analyses were also carried out. This meta-analysis eventually included 11 studies comprising a total 6028 cases and 11488 controls.

**Results:**

There was a significant positive association between long-term mobile phone use (minimum, 10 years) and glioma (OR = 1.44, 95% CI = 1.08–1.91). And there was a significant positive association between long-term ipsilateral mobile phone use and the risk of glioma (OR = 1.46, 95% CI = 1.12–1.92). Long-term mobile phone use was associated with 2.22 times greater odds of low-grade glioma occurrence (OR = 2.22, 95% CI = 1.69–2.92). Mobile phone use of any duration was not associated with the odds of high-grade glioma (OR = 0.81, 95% CI = 0.72–0.92). Contralateral mobile phone use was not associated with glioma regardless of the duration of use. Similarly, this association was not observed when the analysis was limited to high-grade glioma.

**Conclusions:**

Our results suggest that long-term mobile phone use may be associated with an increased risk of glioma. There was also an association between mobile phone use and low-grade glioma in the regular use or long-term use subgroups. However, current evidence is of poor quality and limited quantity. It is therefore necessary to conduct large sample, high quality research or better characterization of any potential association between long-term ipsilateral mobile phone use and glioma risk.

## Introduction

The use of mobile phones has rapidly expanded and increased over the past decade. According to the International Telecommunication Union, the number of mobile phone users over the last decade has increased to a total of 7 billion users worldwide in 2014. Mobile phone users are exposed to radiofrequency electromagnetic fields (EMFs). Such exposure has been associated with increasing concern over potential carcinogenic effects of exposure to the EMF emitted from cellular phones has grown in parallel to increased usage, particularly with regards to a potential increased risk for brain tumors. Glioma is the most common malignant tumor of the central nervous system, and its prognosis is extremely rapid. Glioma may originate from a variety of different aetiological pathways relative to other non-glioma cerebral tumors. However, the exact pathogenesis of glioma remains unclear [1]. Whilst an underlying biological mechanism linking mobile phones use with glioma risk has not been established, the issue remains controversial and topical.

The long latency period between radiation exposure and subsequent tumor development mean that many studies are of insufficient follow-up length to study such potential associations and long-term risk. Whilst, more studies have recently reported an association between long-term (≥ 10 years) mobile phone use and an increase risk of glioma, the absolute number of such studies is small [2]. An observation period of at least 10 years is considered the minimum required to study the long-term carcinogenic risks of RF field exposure during use of mobile phone.

The tendency for users to favour one side of the head when using mobile phones means that the ipsilateral brain is especially exposed to radiation, whereas the contralateral side is much less exposed [3]. Therefore, an important part of researching potential links between mobile phone use and tumor risk is to study a potential correlations between the side of the head predominantly used during phone calls and the position of the glioma within the cerebrum.

Recent, meta-analyses [4–7] have explored the association between mobile phone use and tumors including glioma, meningioma and acoustic neuroma. However, apart from the study by Gong et al [7], these studies did not explicitly analyze glioma based on time, partial laterality and glioma grade. Based on the most recent meta-analysis, we included another three studies [8–10] in the present meta-analysis.

We performed a systematic review of studies on mobile phone use and glioma published by the end of 2015. The objective of this meta-analysis was to explore the potential association between mobile phone use and glioma based on time, partial laterality and glioma grade.

## Materials and methods

We compiled relevant studies according to pre-specified inclusion criteria., A systematic review and meta-analysis by conducted using the Cochrane systematic review method. The results of both systematic review and meta-analysis were presented in accordance to the PRISMA (Preferred Reporting Items for Systematic Reviews and Meta—Analyses) statement.

### Literature search strategy

We searched MEDLINE (PubMed), EMBASE, and the Cochrane Library (1980 to 2016) using the following keywords and MESH terms: “mobile phones/cellular phones/cordless phones” and “brain/intracranial/head tumor/cancer/neoplasm”, or “glioma”. All studies satisfying the selection criteria were extracted. The titles and abstracts were examined as part of the preliminary screening process, and full texts were obtained for articles containing information on mobile phone use and glioma. The references of these included studies were then checked to identify additional relevant publications. Additional studies were further identified by manual search. The search was not limited by publication language.

### Selection criteria

Studies were included in the meta-analysis if they satisfied the following inclusion criteria: (1) an average weekly mobile phone use frequency and at least six months of continuous use; (2) recorded side of head predominantly used; (3) reported glioma pathology; (4) sample size must be stated, odds ratios reported for case-control studies; (5) all digital and/or mobile phone types were included in the analyses; (6) the control group comprises healthy subjects; who are not regularly exposed to radiation from mobile phones or other related sources of electromagnetic radiation. Exclusion criteria: (1) tumor not classified and/or data related to glioma not extracted; (2) Insufficient follow-up time; (3) Where the same patient population was analyzed across multiple studies, only the most recent study was included in the meta-analysis.

### Data extraction

Data was extracted to Excel spreadsheets, including summary information around the study design and data quantifying glioma risk. Studies included in the meta-analysis were evaluated for short-term (mobile phone use < 10 years) and long-term effects (defined as mobile phone use for 10 or more years, where use was defined as an average of at least one call per week). The association between mobile phone use and glioma risk was analyzed across three aspects: 1) mobile phone use duration (short vs. long term); 2) partial laterality and 3) tumor grade. Minimum mobile phone use was defined as weekly use on average for a minimum six continuous months (patients must have had at least 1 year of follow-up post glioma diagnosis).

### Quality of the literature

Two reviewers assessed the quality of the included studies independently using the Newcastle–Ottawa Scale (NOS) [11]. The NOS has three three parameters: selection, comparability, and exposure which are assigned maximum scores of 4, 2 3, respectively. A score of 9 indicates the highest quality. Discrepancies were addressed in consultation with a third reviewer. Studies with scores of at least 6 were considered as high-quality.

### Statistical analyses

Associations between mobile phone use and glioma were estimated by Odds Ratios (ORs) with 95% confidence intervals (95% CIs) using the Woolf method [12] via Rev Man 5.3. For all comparisons, a random effects model was used to estimate pooled ORs across studies using the DerSimonian and Laird method [13]. Heterogeneity between studies included in the meta-analysis was assessed using the Cochran’s Q statistic and the I^2^ statistic [14]. Uncertainty in heterogeneity estimates was quantified by 95% CIs for I^2^ statistic [15]. An I^2^ statistic or 95% CIs ≥50%, it denoted a significant heterogeneity. In addition, the H statistic and 95% CIs were used to support the heterogeneity assessment. A H statistic with a value of 1 denoted no heterogeneity; H < 1.2 indicated homogeneity. If H varied from 1.2 to 1.5 when the 95% CI for H contained 1, heterogeneity was not defined under the inspection level of P < 0.05; if the 95% CI for H did not include 1, heterogeneity was considered present. A H statistic value of >1.5 indicated a significant heterogeneity [16]. Subgroup analyses and sensitivity analyses was undertaken localize the source(s) of any heterogeneity and the meta-analysis re-run after heterogeneity was corrected. We will only make a general description analysis if the reason of heterogeneity is not found. Funnel plots were used to estimate publication bias via Stata 12.0. Egger’s test was used to detect asymmetry of the funnel plots for assessing publication bias. P < 0.05 was considered statistically significant.

## Results

**Fig 1:**
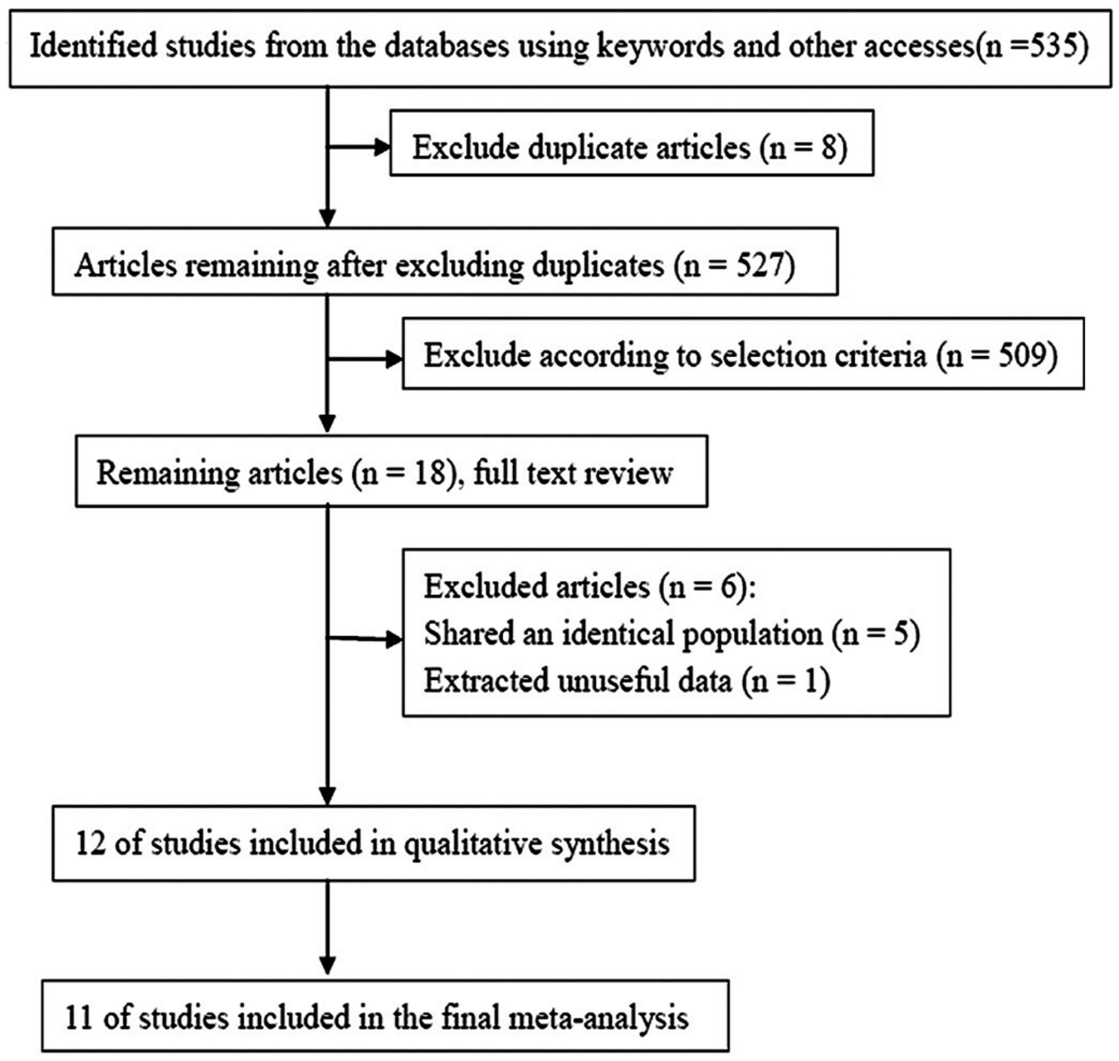
Study flow diagram.

Fig 1 summarizes the process for identifying and screening candidate studies. A total of 535 articles were identified. Eight duplicate articles and an additional 509 articles that did not satisfy the selection criteria were excluded. After reading the full texts of the remaining 18 articles [8–10, 17–31], a further six articles were excluded. A total of 12 articles were included in the qualitative synthesis. Eleven of these including 6028 cases and 11488 controls were included in the meta-analysis (Fig 1). Characteristics of the included studies are summarized in Table 1.

**Table 1:** Eleven studies related to mobile phone use and glioma.

Author, year of publication	Country	Years	No. of cases participation (%)	No. of controls participation (%)
Inskip et al [8](2001)	USA	18–90	782(80)	799(86)
Christensen et al [17](2005)	Denmark	20–69	252(71)	822(64)
Lonn et al [18] (2005)	Sweden	20–69	371(74)	674(71)
Hepworth et al [19] (2006)	English	18–69	966(51)	1716(45)
Schuz et al [20] (2006)	Germany	30–59	366(80)	1535(63)
Klaeboe et al [21] (2007)	Norway	19–69	289(77)	358(69)
Lahkola et al [22] (2007)	5 counties^a^	18–69	1521(60)	3301(50)
Takebayashi et al [23] (2008)	Japan	30–69	88(58.7)	196(52.5)
Hardell et al [24] (2011)	Sweden	20–80	1251(85)	2438(84)
Gaëlle Coureau et al [9] (2014)	France	>16	596(73)	1192(45)
Yoon S et al [10] (2015)	Korea	15–69	285(32)	285(27)

^a^:Denmark, Finland, Norway, Sweden, UK-Southeast England.

### Quality assessment and publication bias

**Fig 2:**
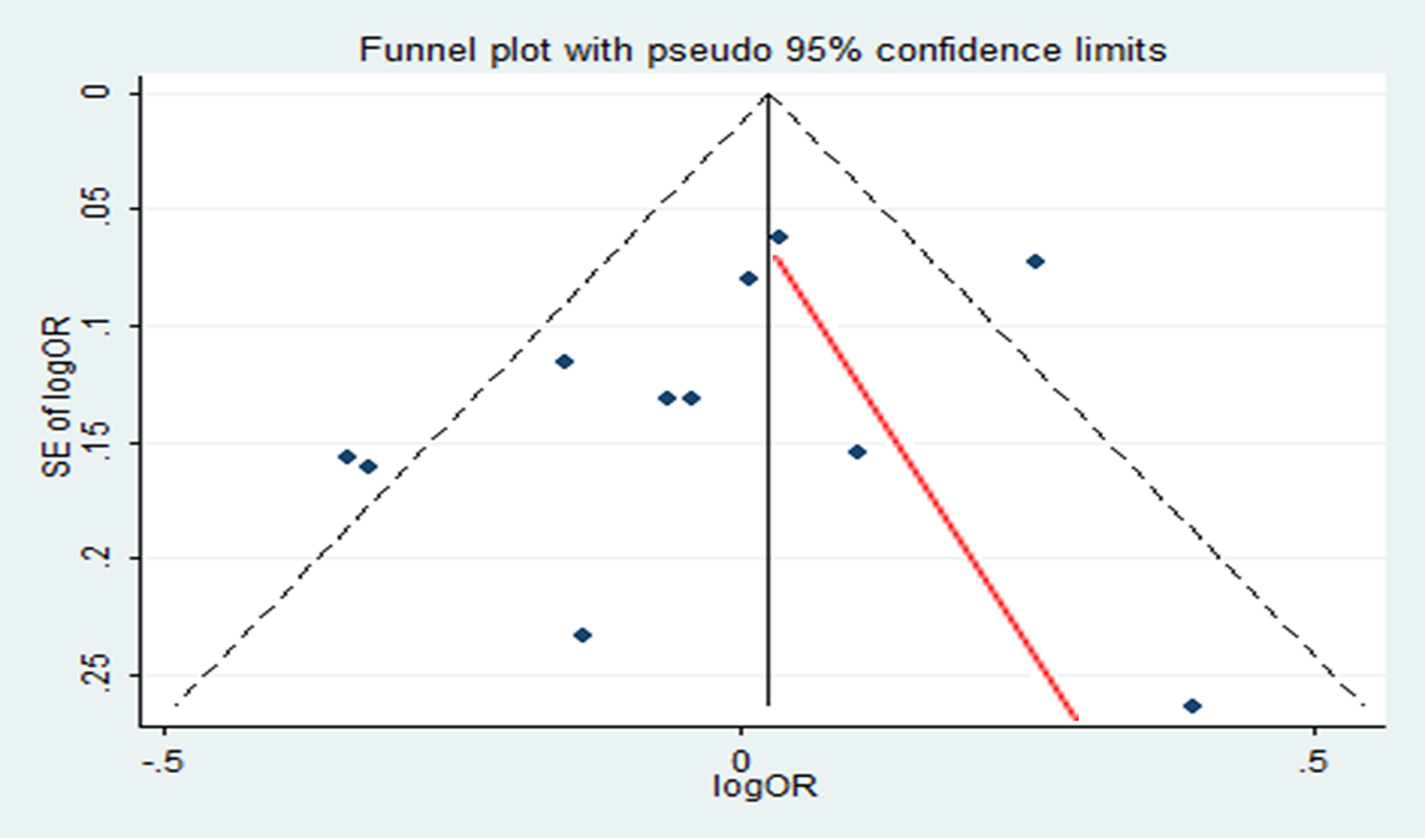
Publication bias for for glioma. OR, odds ratio; SE, standard error.

Table 2 shows the quality assessment for each study. Publication bias was detected by funnel plots and Egger's test. Fig 2 indicates that there was no significant publication bias (Egger’s test, P = 0.218).

**Table 2:** Quality of studies.

study	section				Comparability	Exposure			total
	Is the case definition adequate?	Representativeness of the Cases	Selection of Controls	Definition of Controls		Ascertainment of Exposure	Is same method for case and control?	Nonresponse rate	
Inskip et al [8] (2001)	1	1	1	1	1	1	1	1	8
Christensen et al [17] (2005)	1	1	1	1	2	1	1	1	9
Lonn et al [18] (2005)	1	1	1	1	1	1	1	1	8
Hepworth et al [19] (2006)	1	1	1	1	2	1	1	1	9
Schuz et al [20] (2006)	1	1	1	1	2	1	1	1	9
Klaeboe et al [21] (2007)	1	1	0	0	1	1	1	1	6
Lahkola et al [22] (2007)	1	1	1	1	1	1	1	1	8
Takebayashi et al [23] (2008)	1	1	0	0	2	1	1	1	7
Hardell et al [24] (2011)	1	1	1	1	1	1	1	1	8
Gaëlle Coureau et al [9] (2014)	1	1	1	2	1	1	1	1	9
Yoon S et al [10] (2015)	1	1	0	0	1	1	1	1	7

### Heterogeneity

Table 3 presents the results of the quantification of heterogeneity. Based on the criterion that I^2^ ≥ 50% indicates heterogeneity, we found that mobile phone use and mobile phone use ≥ 10 years both had a I^2^ value ≥ 50%, which indicated substantial heterogeneity. The estimates for mobile phone use and mobile phone use ≥ 10 years were 62% (95% CI 27–80%) and 72% (95% CI 40–87%), respectively.

**Table 3:** Quantification of heterogeneity of pooled estimates.

	I^2^ (95% CI)	H (95% CI)
**Mobile phone use**	62 (27–80)	1.6 (1.2–2.2)
**Mobile phone use≥10 years**	72 (40–87)	1.9 (1.3–2.8)
**Ipsilateral use**	7 (0–73)	1.0 (1.0–1.9)
**Contralateral use**	49 (0–78)	1.4 (1.0–2.2)
**Mobile phone use with low grade glioma**	38 (0–79)	1.3 (1.0–2.2)
**Mobile phone use≥10 years with low grade glioma**	5 (0–90)	1.0 (1.0–3.2)
**Mobile phone use with high grade glioma**	51 (0–84)	1.4 (1.0–2.5)
**Mobile phone use≥10 years with high grade glioma**	19 (0–92)	1.1 (1.0–3.4)

No heterogeneity was observed for ipsilateral and contralateral use. The estimates for ipsilateral and contralateral use were 7% (95% CI 0–73%) and 49% (95% CI 0–78%), respectively. However, as ipsilateral use ≥ 10 years and contralateral use ≥ 10 years were both reported by two studies, the heterogeneity was not quantified.

In the low-grade glioma groups, mobile phone use and mobile phone use ≥ 10 years had similar results, with estimates being 38% (95% CI 0–79%) and 5% (95% CI 0–90%), respectively. In high-grade glioma, there was heterogeneity for the mobile phone use group. The estimate for mobile phone use was 51% (95% CI 0–84%). For mobile phone use ≥ 10 years, the estimate was 19% (95% CI 0–92%), indicating no heterogeneity. The H statistic and 95% CIs were used to replicate these results.

### Mobile phone use and glioma risk

**Fig 3:**
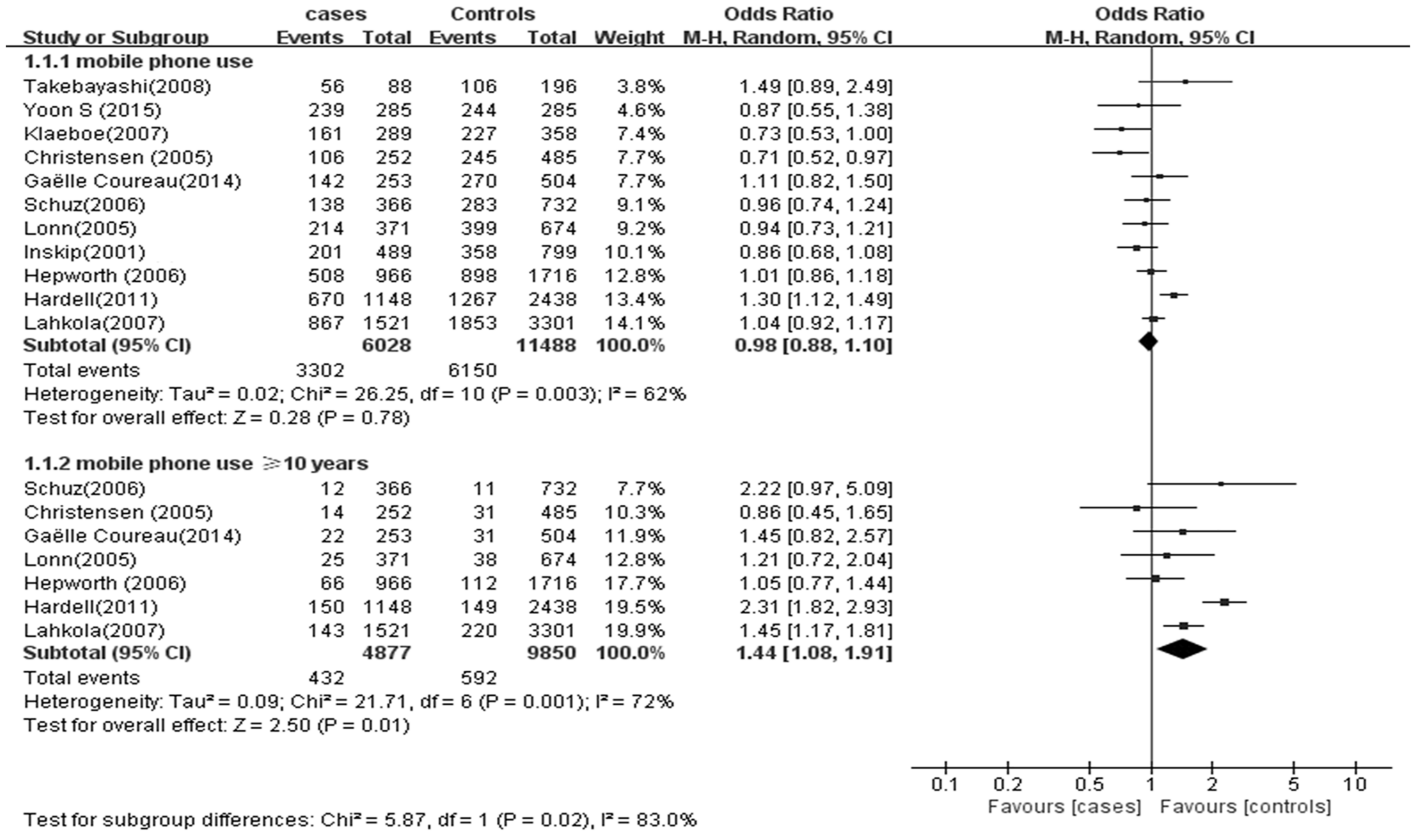
Mobile phone use and the risk of glioma.

Pooled ORs across mobile phone of use any duration were estimated. No association between mobile phone use and glioma risk was observed across the total population (OR 0.98; 95% CI = 0.88–1.10) (Fig 3). However, mobile phone use for ≥10 years was associated with 1.44 times the pooled odds of glioma (OR 1.44; 95% CI = 1.08–1.91) (Fig 3).

### Mobile phone use habit and glioma

**Fig 4:**
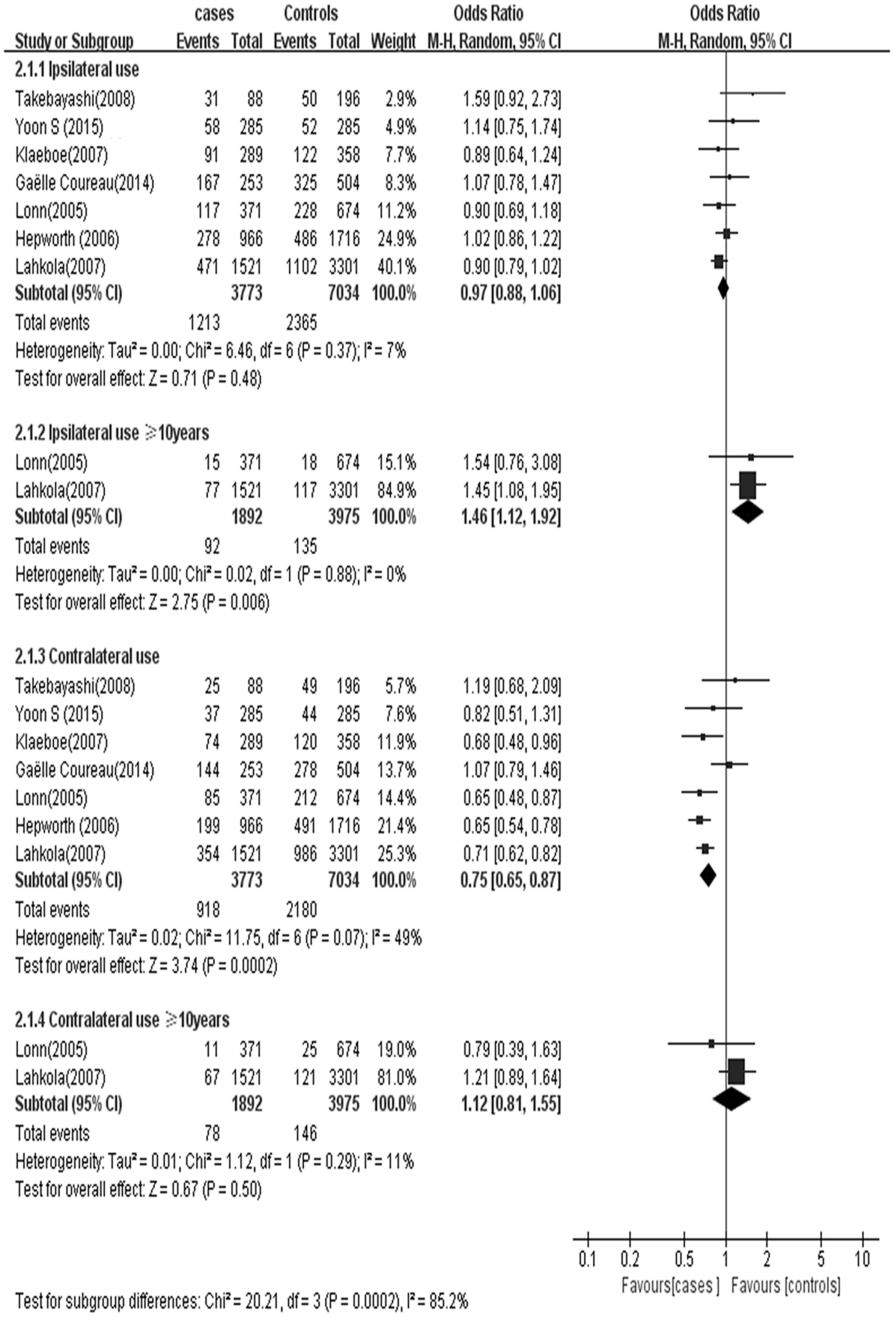
Mobile phone use partial side and the risk of glioma.

A subgroup analysis was conducted dividing subjects by preferred head side use location (ipsilateral and contralateral use) and based on duration of mobile phone use. (Fig 4). Pooled ORs across ipsilateral and contralateral use were estimated to assess the association between mobile phone use habit and glioma. No association was observed in either the ipsilateral use group (OR 0.97; 95% CI = 0.88–1.06), the contralateral use subgroup (OR 0.75; 95% CI = 0.65–0.87) and long-term contralateral use (OR 1.12; 95% CI = 0.81–1.55) (Fig 4). However, long-term ipsilateral use was associated with 1.46 times the odds of glioma (OR 1.46; 95% CI = 1.12–1.92).

### Mobile phone use and glioma grade

**Fig 5:**
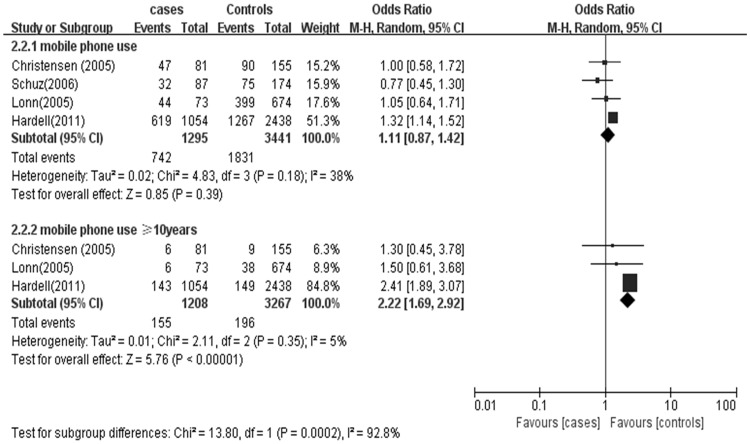
Mobile phone use and the risk of low-grade glioma.

**Fig 6:**
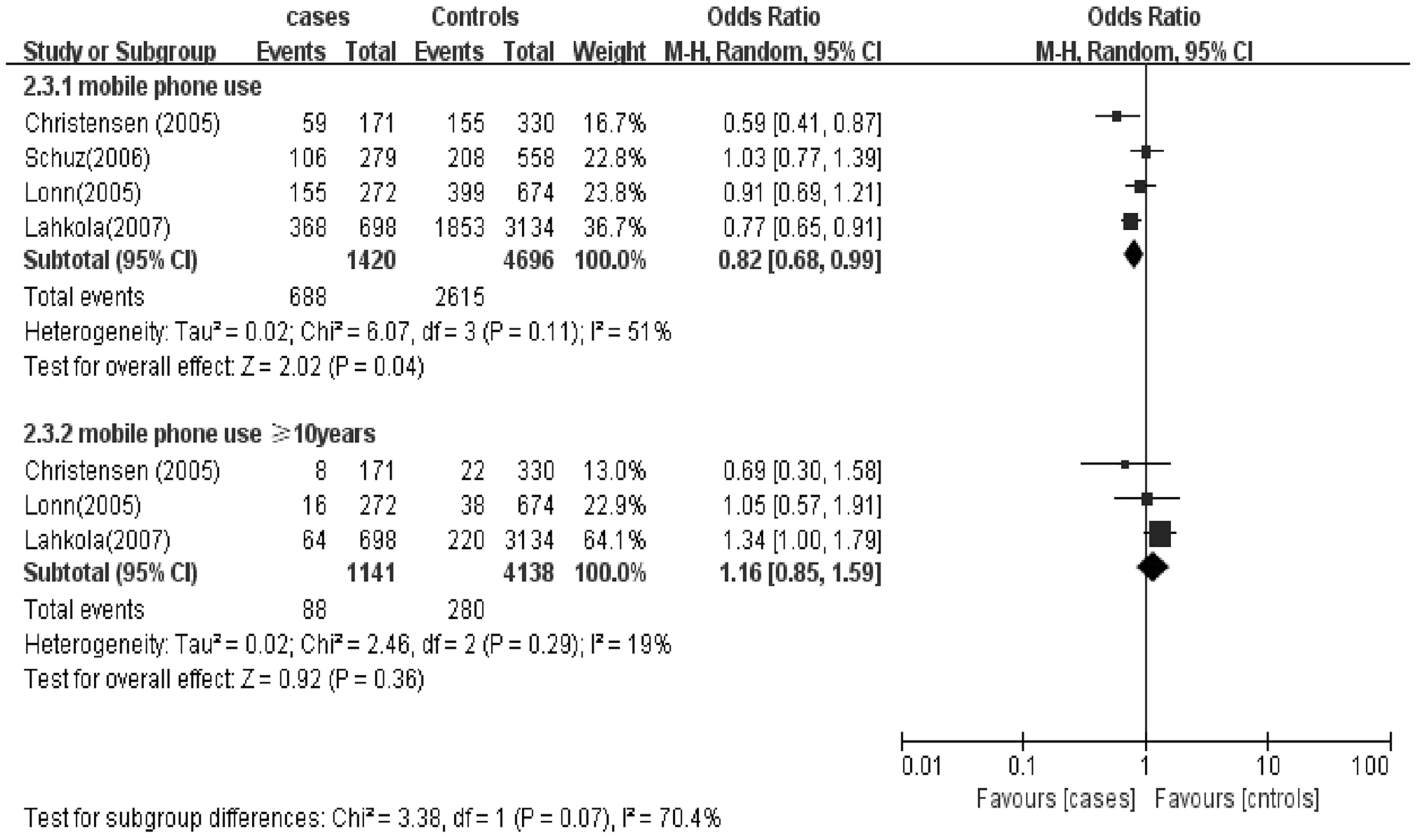
Mobile phone use and the risk of high-grade glioma.

A sub-group analysis was conducted based on both glioma grade (low vs. high) and mobile phone use duration (short-term vs. long-term) (Figs 5 and 6). Pooled ORs were estimated to assess the association between mobile phone use and glioma grade. Mobile phone use was associated with 1.11 times the odds of, specifically, low grade glioma (OR 1.11; 95% CI = 0.87–1.42), but the association was not statistically significant (P > 0.05) (Fig 5). However, there was an association between long-term mobile phone use and low-grade glioma. The signal was larger in that subset of long-term (≥10 years) users with 2.22 times the odds of glioma (OR 2.22; 95% CI = 1.69–2.92). Mobile phone use of any duration was not associated with high-grade glioma (OR 0.82; 95% CI = 0.68–0.99). And no association was observed between long-term use and high grade glioma (OR 1.16; 95% CI = 0.85–1.59) (Fig 6).

## Discussion

In May 2011, Radiofrequency electromagnetic fields (RF-EMF) were classified as carcinogenic to humans (group 2B) by the International Agency for Research on Cancer (IARC). This classification was made in the setting of limited epidemiologic evidence, mechanistic support or animal modelling supporting a potential association between exposure to wireless phones and increased risk of glioma and acoustic neuroma [32]. The most important and comprehensive data included in this study was sourced from the Hardell research group and the international cancer research department. The interphone is the largest case-control study. However, it is accepted widely that the quality is higher and the result is worth referring in Hardell research group according to comparison with the Interphone group by Hardell et al [33]. The objective of this study was to conduct a meta-analysis of available epidemiologic studies of glioma and mobile phone use. While we did not observe correlation between mobile phone use of any duration and glioma risk, there was an association between long-term mobile phone use and glioma. However, the analysis was limited by significant heterogeneity between trials and the paucity of data on long term use (≥10 years).

In the final analysis, we observed that there was a statistical association between long-term mobile phone use and glioma risk, suggesting a possible dose-response relationship. No relationship was observed between risk of glioma and any period ipsilateral use of mobile phone which is consistent with the results of previous meta-analyses [4–7]. However, long-term ipsilateral use was associated with increased risk of glioma. Contralateral use was not associated with glioma regardless of the duration of mobile phone use. These findings suggest a possible role of cumulative exposure and regional localization in a potential underlying biological mechanism links mobile phone use with glioma [34, 35]. Limited data exist supporting ipsi/contralateral mobile phone use with glioma, larger and longer studies are required to better characterize this possible link.

Mobile phone use was associated with increased odds of low grade glioma across both the any period use group and the long-term use subgroup. However, no such association was observed in the any-duration mobile phone use and the long-term use subgroups. Therefore, there is no evidence to suggest that long-term mobile phone use is associated with high-grade glioma. This phenomenon mainly is depended on the biological differences between low-grade glioma and high grade-glioma. Low-grade glioma (WHO grade II tumor) is associated with a long latency period, and is thus potentially more vulnerable to radiation from mobile phones, which in itself may be a chronic carcinogenic agent. Cases affected by radiation are more likely to be detected. Relative to low-grade glioma, high-grade disease has a short latency period. However, most secondary gliomas initially progress from low-grade glioma. Generally, within high grade tumors, WHO grade III glioma has a longer latency period and course of disease than WHO grade IV glioma. However, in most studies, WHO grade III and IV glioma are analyzed together and not separated in order to assess differential effects by grade. Primary glioblastoma progresses fast and has both a short latency period and disease course. These factors may affect the analysis for any association between mobile phone use and high-grade glioma.

However, these results supporting a potential association between mobile phone use and glioma risk need to be interpreted with caution secondary to marked between-trial heterogeneity. The observed correlation between long-term use of mobile phone and glioma risk may be artificial, secondary to systematic differences in response rates between cases and controls.

Our study has a few important limitations. First, in terms of the quality of the literature, there is insufficient primary evidence of the effect only being limited to case–control studies, and the number of cases in a study was insufficient to represent a population. The number of included studies was not large, especially when performing subgroups analysis. Although the subgroup analysis revealed almost no heterogeneity in subgroups analysis, undetected heterogeneity in the Cochrane data still exists [36], especially for small meta-analyses, which may introduce bias. We used statistical testing and graphs to explore publication bias, and eventually detected no publication bias. Even so, the included studies were published in English, which may exclude related studies. Although we searched recent studies, the number of recent studies included in the meta-analysis is too small. For a more convincing review, it is necessary to include the recent studies in the present meta-analysis. Recall bias and selection bias may decrease the quality and reliability of mobile phone exposure data. Differences in recall bias and selection bias, both of which have been described in detail elsewhere [37], may further influence the pooled results. In a validation study of short-term recall of mobile phone use, it was reported that substantial random errors could reduce the power of the INTERPHONE study to detect an increased risk of brain tumor by Vrijheid et al [38]. In addition, age is another limitation, as older patients use would have used mobile phones for a longer time than younger patients. Furthermore, there may be recall bias when older patients state their duration of mobile phone use. Moreover, random errors and selection bias could nominally decrease the estimated risk of brain tumor through using the INTERPHONE data [34]. Furthermore, we did not adjust the analyses for some potential confounding factors in the studies that maybe affect the final result including cognitive impairment and observation bias.

## Conclusions

In conclusion, using meta-analysis, we observed the suggestion of no association between any duration mobile phone use and an increased risk of glioma, but there was an association among long-term users of ≥10. Furthermore, we observed a correlation between the risk of mobile phone use and low-grade glioma in particular, especially long-term users. However, our findings require confirmation with prospective, longer-term studies.

## Supporting information

S1 Table PRISMA NMA checklist for this article. (DOCX)
